# Review of CDC’s Suspension of and Advance Written Approval Process for Dogs Entering the United States from Egypt — May 2019–December 2020

**DOI:** 10.15585/mmwr.mm7134a2

**Published:** 2022-08-26

**Authors:** Michelle Latzer, Emily G. Pieracci, Ashley Altenburger, Kendra E. Stauffer, Clive M. Brown

**Affiliations:** ^1^Division of Global Migration and Quarantine, National Center for Emerging and Zoonotic Infectious Diseases, CDC; ^2^Oak Ridge Institute for Science and Education, Oak Ridge, Tennessee; ^3^Animal and Plant Health Inspection Service, U.S. Department of Agriculture, Riverdale, Maryland.

Dog-maintained rabies virus variant (DMRVV) was eliminated in the United States in 2007. During 2015–2019, three dogs with rabies were imported into the United States from Egypt, where DMRVV is endemic. CDC developed a risk mitigation strategy, in consultation with a diverse group of subject matter experts, that permitted 296 dogs to be imported from Egypt during May 10, 2019–December 31, 2020, minimizing the risk for future rabid dog importations. The broadly vetted risk mitigation strategy, which included serologic testing for rabies antibody titer, improved CDC’s ability to ensure that imported dogs from Egypt posed no public health risk in the United States. This strategy could be used to guide future policy decisions regarding dog importations.

Rabies is responsible for an estimated 59,000 human deaths annually worldwide; 98% of these deaths are attributed to bites from rabid dogs (*1*). Although numerous variants of the rabies virus exist, DMRVV is of greatest concern because of its global presence in unvaccinated dog populations (*1*). The endemicity of DMRVV in approximately 110 countries creates a risk that DMRVV could be reintroduced into the United States (*2*). Rabies virus is usually transmitted through saliva from the bite or scratch of an infected animal (*3*). The incubation period in dogs and humans is variable, but most dogs infected with the rabies virus begin to show clinical signs of disease within 1–3 months of exposure (*4*). Rabies is nearly 100% fatal in both humans and animals after clinical signs appear. However, routine rabies vaccination in dogs is nearly 100% effective in preventing rabies infection. Hence, the United States requires that all dogs from rabies endemic countries be vaccinated against rabies before importation.

Since 2015, three dogs with confirmed rabies have been exported from Egypt into the United States (*5*–*7*). Molecular characterization confirmed that the DMRVV known to circulate in Egypt was present in each dog, suggesting that the dogs were infected with DMRVV in Egypt before entering the United States. The repeated export of rabid dogs from Egypt in 2015, 2017, and 2019 suggests that challenges might exist with canine rabies control within the country; these challenges might include poor vaccine quality, improper vaccine storage or administration, inaccurate record keeping, and general lack of oversight from veterinary authorities within the country. With each instance of DMRVV importation into the United States, many persons and animals receive postexposure prophylaxis and undergo monitoring and assessment by their state or local health departments, resulting in costs of ≥$200,000 per event (*8*).

As a result of the public health threat posed by dogs imported from Egypt, a suspension of dogs entering the United States from Egypt was issued on May 10, 2019*. Recognizing that returning citizens, including military service members, might be importing their dogs into the United States, CDC developed and implemented a risk mitigation strategy to minimize the likelihood of importing DMRVV from Egypt during the suspension.

Data for the current report were collected through the CDC Application for Permission to Import A Dog Inadequately Immunized Against Rabies — Single Entry forms^†^. Applications were uploaded and stored in CDC’s Quarantine Activity Reporting System (QARS), a secure database that records CDC’s border public health activities, including actions taken for CDC-regulated importations. Application data were deidentified for analysis before being extracted from QARS for analysis. This activity was reviewed by CDC and was conducted consistent with applicable federal law and CDC policy.^§^

Dogs were defined as inadequately immunized if they received a rabies vaccine not licensed for use in dogs in the United States, or if they were vaccinated by a veterinarian not state-licensed in the United States, because this is considered unverifiable documentation. Before importation, owners of inadequately vaccinated dogs were required to submit rabies antibody serologic test results from a laboratory approved by the World Organisation for Animal Health (WOAH). If serologic test results were >0.5 IU/mL, CDC issued a conditional import permit, which required revaccination with a rabies vaccine licensed by the U.S. Department of Agriculture within 10 days of arrival in the United States. Under the risk mitigation strategy, dogs entering the United States were required to be adequately protected against rabies and comply with recommendations outlined in the National Association of State Public Health Veterinarians Rabies Compendium (*7*) through the conditional permit process, which required dogs to be revaccinated upon arrival. This strategy was developed through consultation with rabies subject matter experts from federal and state agencies, CDC policy experts, and the U.S. Department of Health and Human Services (HHS) Office of the General Counsel.

To prevent the import of rabid dogs into the United States, a working group consisting of state and federal partners, animal importation experts, and rabies subject matter experts was convened to discuss processes to reduce the possibility of DMRVV importation from countries with endemic DMRVV, in consultation with CDC policy experts and HHS Office of the General Counsel. The group reviewed rabies epidemiologic data from multiple sources and compared current U.S. dog importation requirements with those of other DMRVV-free countries. The group discussed critical data elements to include in the risk mitigation algorithm (Figure). The meetings also provided an opportunity for federal and state partners to voice their concerns and propose long-term solutions to address the heightened possibility of DMRVV importation from Egypt. Consultations with rabies and animal importation experts from the European Union, the Pan American Health Organization, and the Veterinary Border Inspection Office, Norwegian Food Safety Authority also contributed to the working group’s deliberations.

**FIGURE:**
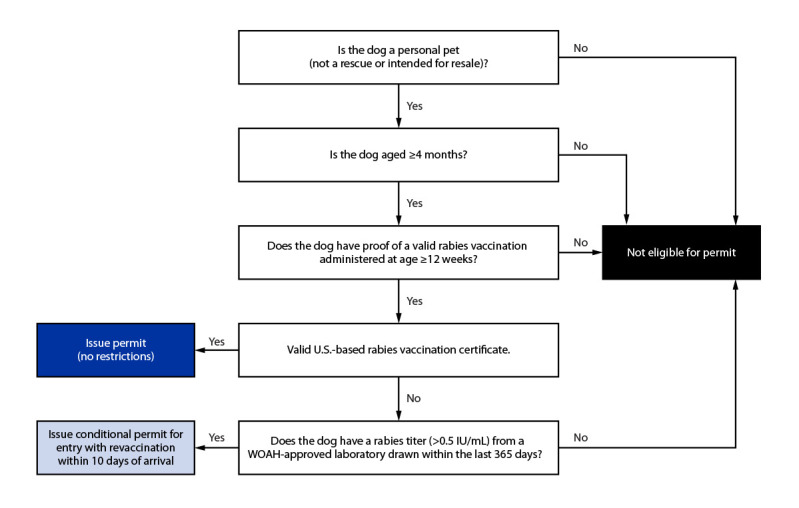
Risk mitigation algorithm implemented following May 10, 2019 suspension of dog importations from Egypt — United States, May 10,2019–December 31,2020 **Abbreviations**: RVC = rabies vaccination certificate; WOAH = World Organisation for Animal Health.

Consensus was achieved on the following processes: persons wishing to import a dog from Egypt were required to apply for and receive a CDC dog importation permit; in addition eligible owners or importers working on behalf of an owner were required to submit 1) CDC Application for Permission to Import a Dog Inadequately Immunized Against Rabies form; 2) proof of a current, valid rabies vaccination certificate;^¶^ 3) a rabies serologic test result of >0.5 IU/mL from a WOAH-approved laboratory when the dog was considered to be inadequately immunized against rabies; 4) evidence that the dog was aged ≥4 months and eligible for entry into the United States;** 5) verifiable identification information (microchip); and 6) documentation of the owner’s employment, university, or other evidence indicating relocation or return to the United States.

During May 2019–December 2020, permits for the importation of 296 dogs from Egypt into the United States were issued (Table 1). None of the 296 dogs developed rabies after importation. Among the applicants, 42% were short-term travelers returning to the United States after vacationing in Egypt and 50.0% had dogs that had been vaccinated outside the United States (Table 2). The average processing time for permit requests was 7.9 days for U.S.-vaccinated dogs and 10.4 days for foreign-vaccinated dogs. 

**TABLE 1:** Permits issued to imported dogs from Egypt during the suspension — CDC, May 10, 2019–December 31, 2020

Characteristic	No. (%)
**No. of permits (% of total)**	
**Port of entry**	
Hartsfield-Jackson Atlanta International Airport	14 (4.7)
O’Hare International Airport	15 (5.1)
Detroit Metropolitan Wayne County Airport	3 (1.0)
Dulles International Airport	60 (20.3)
George Bush Intercontinental Airport	14 (4.7)
Los Angeles International Airport	18 (6.1)
Miami International Airport	7 (2.4)
Minneapolis-St. Paul International Airport	4 (1.4)
Newark Liberty International Airport	3 (1.0)
John F. Kennedy International Airport	105 (36.8)
Philadelphia International Airport	1 (0.3)
San Francisco International Airport	11 (3.7)
Seattle-Tacoma International Airport	7 (2.7)
Other*	29 (9.8)
**Total**	**296 (100.0)**
**Entry method**	
Hand carried	143 (48.3)
Checked baggage	102 (34.5)
Cargo	42 (14.2)
Land border	9 (3.0)
**Total**	**296 (100.0)**
**Dog category/Importer affiliation**	
Military working dog	
U.S. Department of Defense	13 (4.4)
Personal pet	
U.S. Department of Defense	14 (4.7)
U.S. Department of State	35 (11.8)
Nongovernment contractors	35 (11.8)
University employees	17 (5.7)
Travelers in Egypt <1 year	127 (42.9)
Other†	55 (18.6)
**Total**	**296 (100.0)**

*Ports of entry not staffed by CDC personnel.

† Travelers in Egypt >1 year; first time moving to the United States for work or school.

**TABLE 2:** Number of permits* issued and number needing rabies serology to import dogs from Egypt during suspension, by time of arrival — CDC, May 10, 2019–December 31, 2020

Arrival year and month	No. (%) of permits issued	No. (%) of permits needing rabies serology
**2019**		
May	4 (1.4)	3 (75.0)
Jun	15 (5.1)	3 (20.0)
Jul	18 (6.1)	6 (33.0)
Aug	27 (9.1)	11 (41.0)
Sep	21 (7.1)	6 (29.0)
Oct	16 (5.4)	10 (63.0)
Nov	20 (6.8)	12 (60.0)
Dec	9 (3.0)	5 (56.0)
**2019 total**	**130 (44.0).**	**56 (37.8)**
**2020**		
Jan	28 (9.5)	16 (57.0)
Feb	13 (4.4)	8 (62.0)
Mar	11 (3.7)	5 (46.0)
Apr	14 (4.7)	6 (43.0)
May	10 (3.4)	8 (80.0)
Jun	10 (3.4)	7 (70.0)
Jul	16 (5.4)	10 (63.0)
Aug	15 (5.1)	6 (40.0)
Sep	12 (4.1)	6 (50.0)
Oct	12 (4.1)	6 (50.0)
Nov	11 (3.7)	7 (64.0)
Dec	14 (4.7)	7 (50.0)
**2020 total**	**166 (56.0)**	**86 (58.1)**
**Overall total**	**296 (100.0)**	**148 (50.0)**

*Excludes 40 applicants who changed their arrival dates after applying for permits and were reissued permits later.

## Discussion

The goals of the risk mitigation strategy were to maximize public health protection, reduce the possibility of DMRVV importation events, minimize the difficulties that importers might face when attempting to import a dog, align with state vaccination requirements, and reduce the costs faced by state government health agencies. Data from this analysis indicated that 50% of dogs imported from Egypt during May 2019–December 2020 were vaccinated outside the United States and might have posed a public health risk if CDC had not required the importers to submit pre-arrival rabies serologic test results and agree to postarrival revaccination of their dogs. The risk mitigation strategy improved CDC’s ability to ensure that these dogs posed no public health risk in the United States. After attempts to import ineligible dogs during the COVID-19 pandemic, CDC temporarily suspended the entry of dogs into the United States from all countries considered high-risk for canine rabies on July 14, 2021.^††^ This suspension used the risk mitigation strategy described in this report as a basis for the temporary CDC dog importation suspension issued in 2021, which uses a combination of import permits, pre-arrival serologic tests, postarrival revaccination, and quarantine (only available during the 2021 suspension). 

The reintroduction of DMRVV in Texas in the 1980s led to a large-scale elimination effort by federal and state public health partners for decades. During that time, DMRVV was associated with the death of two persons (*9*) and approximately $25 million in elimination costs (*10*). Although DMRVV has been eliminated from the United States since 2007, DMRVV has a strong potential to adapt to new hosts, including novel reservoir species (*1*). Potential outcomes of importing rabid dogs include the reintroduction and sustained transmission of DMRVV among domestic animals and wildlife, high costs to eliminate DMRVV from animal populations, and the infection of humans and animals resulting in death. The risk mitigation strategy developed during the 2019–2020 suspension of dogs imported from Egypt allowed for the safe entry of some dogs and prevented transmission of rabies by minimizing the likelihood of introducing DMRVV from Egypt during that period. This strategy could be used to guide future policy decisions regarding dog importations.

**Summary**

**What is already known about this topic?**

Dog-maintained rabies virus variant (DMRVV) was eliminated from the United States in 2007. During 2015–2019, three rabid dogs were imported into the United States from Egypt, where DMRVV is endemic.

**What is added by this report?**

Consultation with subject matter experts enabled CDC to develop a risk mitigation strategy that permitted 296 dogs to be imported from Egypt during May 10, 2019–December 31, 2020, and reduced the risk for rabid dog importations.

**What are the implications for public health practice?**

The risk mitigation strategy improved CDC’s ability to ensure that imported dogs posed no public health risk in the United States. This strategy could be used to guide future policy decisions regarding dog importations.

## Footnotes

*
https://www.federalregister.gov/documents/2019/05/10/2019-09654/notice-of-temporary-suspension-of-dogs-entering-the-united-states-from-egypt

†
https://omb.report/icr/202203-0920-014/doc/119915200

§ 45 C.F.R. part 46, 21 C.F.R. part 56; 42 U.S.C. Sect. 241(d); 5 U.S.C. Sect. 552a; 44 U.S.C. Sect. 3501 et seq.

¶ Issues with vaccine quality and administration raise questions about the validity of dogs’ foreign rabies vaccination certificates; these dogs are considered inadequately immunized.

** The age of dogs entering the United States is verified by licensed U.S. veterinarians using dentition eruption patterns of deciduous and permanent teeth.

††
https://www.federalregister.gov/documents/2021/06/16/2021-12418/temporary-suspension-of-dogs-entering-the-united-states-from-high-risk-rabies-countries
